# Accelerated Long-Term Forgetting Can Become Apparent Within 3–8 Hours of Wakefulness in Patients With Transient Epileptic Amnesia

**DOI:** 10.1037/neu0000114

**Published:** 2014-08-04

**Authors:** Serge Hoefeijzers, Michaela Dewar, Sergio Della Sala, Christopher Butler, Adam Zeman

**Affiliations:** 1 Human Cognitive Neuroscience, Department of Psychology and Centre for Cognitive Ageing and Cognitive Epidemiology, University of Edinburgh; 2 Human Cognitive Neuroscience, Department of Psychology University of Edinburgh and Department of Psychology, School of Life Sciences, Heriot-Watt University; 3 Human Cognitive Neuroscience, Department of Psychology and Centre for Cognitive Ageing and Cognitive Epidemiology, University of Edinburgh; 4 Nuffield Department of Clinical Neurosciences, University of Oxford; 5 Cognitive and Behavioural Neurology, University of Exeter Medical School

**Keywords:** accelerated long-term forgetting, transient epileptic amnesia, memory, consolidation, epilepsy

## Abstract

***Objective:*** Accelerated long-term forgetting (ALF) is typically defined as a memory disorder in which information that is learned and retained normally over standard intervals (∼30 min) is forgotten at an abnormally rapid rate thereafter. ALF has been reported, in particular, among patients with transient epileptic amnesia (TEA). Previous work in TEA has revealed ALF 24 hr - 1 week after initial memory acquisition. It is unclear, however, if ALF observed 24 hr after acquisition reflects (a) an impairment of sleep consolidation processes taking place during the first night’s sleep, or (b) an impairment of daytime consolidation processes taking place during the day of acquisition. Here we focus on the daytime-forgetting hypothesis of ALF in TEA by tracking in detail the time course of ALF over the day of acquisition, as well as over 24 hr and 1 week. ***Method:*** Eleven TEA patients who showed ALF at 1 week and 16 matched controls learned 4 categorical word lists on the morning of the day of acquisition. We subsequently probed word-list retention 30 min, 3 hr, and 8 hr postacquisition (i.e., over the day of acquisition), as well as 24-hr and 1-week post acquisition. ***Results:*** ALF became apparent in the TEA group over the course of the day of acquisition 3–8 hr after learning. No further forgetting was observed over the first night in either group. ***Conclusions:*** The results of this study show that ALF in TEA can result from a deficit in memory consolidation occurring within hours of learning without a requirement for intervening sleep.

Accelerated long-term forgetting (ALF) is usually defined as a memory disorder in which information that is apparently learned and retained normally over standard intervals in neuropsychological testing (∼30 min), is forgotten at an abnormally rapid rate thereafter (Butler & Zeman, 2008). ALF has been reported in patients with temporal lobe epilepsy (TLE; e.g., Blake, Wroe, Breen, & McCarthy, 2000; Jansari, Davis, McGibbon, Firminger, & Kapur, 2010; Kapur et al., 1997; Kemp, Illman, Moulin, & Baddeley, 2012; Lucchelli & Spinnler, 1998; Martin et al., 1991; Mayes et al., 2003; McGibbon & Jansari, 2013; O’Connor, Sieggreen, Ahern, Schomer, & Mesulam, 1997; Wilkinson, Holdstock, Baker, Herbert, Clague, & Downes, 2012; for an exception, see Giovagnoli, Casazza, & Avanzini, 1995), in particular transient epileptic amnesia (TEA; Butler et al., 2007, 2009; Manes, Graham, Zeman, de Lujan Calcagno, & Hodges, 2005; Muhlert, Milton, Butler, Kapur, & Zeman, 2010).

TEA, thought to be a subtype of TLE (Butler et al., 2007; Zeman & Butler, 2010), is characterized by brief, recurrent episodes of transient amnesia occurring as a result of epilepsy. During these episodes, declarative memory becomes impaired while other cognitive functions remain intact. The episodes are usually abolished by anticonvulsant treatment. However, around 50% of patients with TEA report additional, interictal ALF that remains symptomatic and measurable even after successful treatment of the amnestic episodes (Butler et al., 2007; Zeman, Butler, Muhlert, & Milton, 2013). Existing evidence suggests that ALF in TEA is associated primarily with impairment of memory consolidation rather than with impairments of acquisition or retrieval: TEA patients continue to demonstrate ALF even when their performance during acquisition is matched with that of controls, using recognition tests that facilitate retrieval (Butler et al., 2007; Butler, Kapur, Zeman, Weller, & Connelly, 2012; Hoefeijzers, Dewar, Della Sala, Zeman, & Butler, 2013; Muhlert et al., 2010).

The precise time course of ALF is, however, uncertain. Experimental tasks using extended retention intervals have revealed increased forgetting rates in TEA over intervals varying from 24 hr to 6 weeks (Butler et al., 2007, 2009; Manes et al., 2005; Muhlert et al., 2010), but it remains to be established whether ALF in TEA is apparent over delays shorter than 24 hr. Determining the time course of ALF should shed light on its underlying mechanism. Two main possibilities have previously been proposed.

ALF in TEA could be associated with *impaired sleep-consolidation processes* (Muhlert et al., 2010; Zeman & Butler, 2010), resulting in the appearance of ALF on the day after acquisition, that is, after a period of nocturnal sleep (Muhlert et al., 2010). Indeed, the amnesic attacks experienced by TEA patients often occur on waking, and this nocturnal seizure activity provides a potential mechanism for ALF in TEA (Butler & Zeman, 2008). Alternatively, ALF in TEA could be associated with *impaired daytime-consolidation processes*, resulting in the appearance of ALF over the course of the day of acquisition. A recent study by Deak, Stickgold, Pietras, Nelson and Bubrick (2011) supports an explanation of the latter type for ALF seen in patients with TLE. These authors observed ALF when learning was followed by a 12-hr period of daytime wakefulness, but not when learning was followed by a 12-hr period of nocturnal sleep. Similarly, a recent single-case study (McGibbon & Jansari, 2013) revealed accelerated forgetting within an hour in a patient with ALF in the context of TLE.

In the present study, we examined the “daytime forgetting” hypothesis of ALF in TEA, asking *whether* ALF becomes apparent across the day of acquisition, and, if so, *when* it becomes apparent. To this end, a group of TEA patients who showed ALF after 1 week and a group of matched controls learned four categorical word lists and were asked to recall these at four test intervals: 30 min, 3 hr, 8 hr and 24 hr postlearning. To minimize potential masking of ALF by repeated testing of the same list, we probed a different word list at each test interval. Moreover, in line with the more traditional 1-week ALF test (e.g., Butler et al., 2007), participants were asked to recall all four lists again after 1 week, followed immediately by a yes/no word-recognition test.

## Materials and Method

### Participants

We recruited 17 patients with TEA and 18 controls for this study. All patients met diagnostic criteria for TEA (Zeman, Boniface, & Hodges, 1998): (a) a history of recurrent, witnessed episodes of transient amnesia; (b) intact cognitive functions (aside from memory) during typical episodes, as judged by a reliable witness; (c) evidence for a diagnosis of epilepsy based on one or more of the following: epileptiform abnormalities on an electroencephalogram (EEG), concurrent onset of other clinical features of epilepsy (such as lip smacking or olfactory hallucinations), clear-cut response to anticonvulsant therapy. Moreover, all TEA patients complained of ALF, were on anticonvulsant monotherapy and, at the time of testing, had been free from overt seizures for at least 6 months.

One patient and two controls could not complete the study due to unavailability and were therefore removed from the study. Five TEA patients showed no objective evidence of ALF at 1 week on our experimental test (scores within 1 *SD* of the mean of controls). Because our examination of the early time course of ALF was contingent on the presence of objectively defined ALF using our experimental test, these five patients were also removed from the study. Therefore, the present study included a sample of 11 TEA patients in whom ALF was observed objectively after 1 week, and a sample of 16 controls.

Table 1 gives clinical information for the patients with TEA, including the grounds for the diagnosis of TEA.[Table-anchor tbl1]

The TEA patients and controls were matched for age and education (see Table 2). Both patients and controls underwent neuropsychological screening: the National Adult Reading Test (NART; Nelson, 1982) and the Wechsler Abbreviated Scale of Intelligence (WASI) similarities and matrix reasoning subtests (Wechsler, 1999) were used to assess general intelligence. The Wechsler Memory Scale–III (WMS-III) logical memory test (immediate and 30-min delayed recall of Story A only; Wechsler, 1997) and delayed recall of the Rey–Osterrieth complex-figure test (Osterrieth & Rey, 1994) were used as anterograde memory measures. The copy of the Rey–Osterreith complex-figure test also was applied as a measure of visuospatial perception. A test of verbal fluency, the FAS letter fluency test (Spreen & Benton, 1977; Tombaugh, Kozak & Rees, 1999) was used to examine executive function. Last, mood was assessed using the Hospital Anxiety and Depression Scale (HADS; Zigmond & Snaith, 1983). As shown in Table 2, the TEA patients performed well on all psychometric tests administered. The control group outperformed the patient group in the NART, resulting in a subtle, albeit significant group difference in NART-predicted verbal IQ, *t*(25) = −2.076, *p* = .048, *r* = .38 (see Table 2). We discuss this difference further in the results and discussion. Moreover, the TEA group’s HADS score was higher than that of the controls, resulting in a significant group difference, *t*(25) = 2.273, *p* = .032, *r* = .41.[Table-anchor tbl2]

All participants spoke English as their first language and had no symptoms of psychiatric disturbance.

The study was approved by the National Health Service (NHS) Scotland A Research Ethics Committee and by the Psychology Research Ethics Committee of the University of Edinburgh. Informed consent was obtained from each participant according to the Declaration of Helsinki (World Medical Association, 2001).

### Stimuli

Four categorical word lists were designed entitled “Animals,” “City,” “Nature,” and “Groceries.” Each list consisted of 16 category-related words, and all lists were matched for British word frequency (spoken and written) using the British National Corpus (BNC) database (BNC website: http://www.natcorp.ox.ac.uk/corpus/index.xml), and for the psycholinguistic measures familiarity, imaginability, concreteness, and number of letters using the Medical Research Council (MRC) Psycholinguistic database (MRC website: http://websites.psychology.uwa.edu.au/school/MRCDatabase/uwa_mrc.htm). Pilot work confirmed that the four categorical lists were matched for word-list learning over two trials and for recall performance 30 min and 1 week after acquisition.

### Procedure

**Figure 1 fig1:**
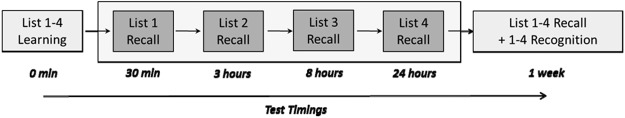
Test procedure. Participants were presented twice with four categorical word lists (16 words per list) entitled “Animals,” “City,” “Nature,” and “Groceries.” List retention was probed 30 min, 3 hr, 8 hr, and 24 hr after word-list learning. To minimize potential masking of ALF by repeated testing of the same list, participants had to recall a different word list at each test interval. One week after word-list learning, participants were asked to recall all four lists again, and this was followed by a yes/no word-recognition test.

Testing took place at the participants’ homes during five sessions (see Figure 1). The experiment began between 9:30 and 10:30 a.m. on Day 1.[Fig-anchor fig1]

#### Word-list learning

The Animals, City, Nature, and Groceries word lists were presented visually and one by one via a 15-inch laptop screen. The order of categorical lists was counterbalanced across participants. Each categorical list was followed directly by an immediate recall test for that list. Following immediate recall of the fourth word list, the word-learning sequence was repeated in the same order, resulting in two learning trials per categorical word list.

During list presentation, each word was presented for 2 s, followed by a fixation cross for 1 s between words. To ensure that participants attended to all presented words, they were asked to read each word aloud. Participants were instructed to remember as many list words as possible for subsequent immediate recall of that list. They were free to recall the words in any order and were asked to indicate when they had completed their recall. Following immediate recall of each list, participants were asked to perform a backward subtraction task for 20 s before presentation of the next list to separate the word lists during learning.

#### Delayed recall

Participants’ retention of the learned word-list material was tested after four intervals: 30 min, 3 hr, 8 hr, and 24 hr after word-list learning (see Figure 1). To minimize potential masking of ALF by repeated testing of the same list, participants had to recall a different word list at each test interval. At all test intervals, participants were tested in the same location in the same room as during word-list learning.

Lists were probed in the counterbalanced order in which they had been presented during list learning. Prior to recall, the title of the to-be-remembered categorical word list (Animals, City, Nature, or Groceries) was presented on the laptop screen as a recall cue. As at immediate recall, participants were free to recall the words in any order, and they were asked to indicate when they had completed their recall.

Throughout the 30-min delay subsequent to word-list learning, participants were presented with a set of 300 complex, everyday-life photos, which they were asked to look at carefully and try to remember for an unrelated memory test. This task formed part of a different study and acted merely as a filler task in the present experiment. The experimenter remained with the participants until the 3-hr delay interval tests were completed. During the 3–8-hr interval, the experimenter was sometimes present, sometimes not, according to the convenience of participants. There was no systematic difference between testing of TEA patients and controls.

One week after word-list learning, participants were asked to recall all four lists again using the categorical titles as recall cues. Thereafter, a yes/no word-recognition test was conducted. In this test, the 16 original words (i.e., the targets) of each list were intermixed with 16 related foils. Thus in total, the recognition test consisted of 64 target items and 64 foils. Within each category, we manipulated the relatedness between targets and foils, such that six foils were semantically distant—phonetically related; five foils were semantically close—phonetically unrelated and five foils were semantically distant—phonetically unrelated. For example, in the case of the Animals categorical word list, “mouse” was used as a foil for “moose” (semantically distant—phonetically related), “lion” was used as a foil for “tiger” (semantically close—phonetically unrelated), and “snake” was used as a foil for “bear” (semantically distant—phonetically unrelated).

As during word learning, words were presented visually on a laptop screen. For each word, participants had to indicate verbally whether or not they had been presented with that word 1 week earlier.

Participants were not informed about the delayed memory tests. However, after completion of the 8-hr interval testing session, they were explicitly asked not to think about any of the tests they had undertaken during that day.

### Test Scoring

#### Word-learning performance

Because there were no significant differences between categorical lists at either learning trial (see online Supplementary Data 1 for scores and *p* values), we computed an average list score for learning Trial 1 and learning Trial 2, that is, average number of words recalled correctly per learning trial per participant.

#### Delayed recall

We computed percentage retention scores for each participant for each delay interval. Percentage retention scores control for potential individual and group differences as well as any interlist variation at immediate recall. The latter was important because a different category list was probed at each delay interval. We calculated the percentage retention scores by dividing the number of words recalled after a given delay by the number of words recalled for that specific word list at learning Trial 2, and multiplying the quotient by 100 [(recall score after delay/recall score for that specific list at learning Trial 2) × 100]. Because all four lists were probed after 1 week and at learning Trial 2, we computed an average list-retention score for each participant for the 1-week word-recall test.

#### Word-recognition test

Calculations of *d*’ values were made from hit and false-alarm rates: *z*(hit rate) – *z*(false-alarm rate). Hit rate refers to the number of original stimuli identified as “Old”, divided by the total number of original stimuli presented in the recognition test. False-alarm rate refers to the number of foils identified as “Old”, divided by the total number of foils presented in the recognition test.

#### Forgetting rates

Forgetting rates were list-specific in that a forgetting score was computed for each learned list from learning Trial 2 to one of the 4 early delay intervals (early forgetting), and from that early delay interval to the 1-week delay interval (late forgetting). The forgetting rates over the early (≤24 hr) and late (1 week) delay intervals were calculated as

*Early forgetting* = (learning Trial 2 recall score – recall score at specific delay interval)/(learning Trial 2 recall score) × 100.

*Late forgetting* = (recall score at specific delay interval – 1-week recall score)/(recall score at specific delay interval) × 100.

#### Guess correction

The use of categorical word lists and cued-recall tests can raise the tendency for participants to guess (see, e.g., Huff, Meade, & Hutchison, 2011; Tulving & Pearlstone, 1966). Therefore, in addition to analyzing raw retention scores, we also analyzed “guess-corrected” scores. We did so by applying a guess correction to word-list recall during learning and delayed recall, followed by computation of guess-corrected percentage retention scores. Word recall was corrected by dividing the number of correct words recalled by the total number of category-related words recalled, and multiplying this quotient by the number of correct words recalled: correct recalls × correct recalls/(correct recalls + incorrect recalls). For example, if 8 out of the 10 recalled category words were included in the presented categorical word list, the guess-correction factor would be 8/(8 + 2) = 0.8, resulting in a corrected recall score of 8 × 0.8 = 6.4.

### Statistical Analyses

We applied a combination of independent *t* tests and mixed-factors ANOVAs to examine memory scores across the delay intervals in the two groups. Planned comparisons were carried out between pairs of delay intervals (30 min, 3 hr, 8 hr, and 24 hr) to examine changes in retention over these intervals. We applied Pearson correlations to examine associations between forgetting rates over the early (≤24-hr) and late (1-week) delay intervals, as reported previously (Butler et al., 2012; Hoefeijzers et al., 2013; Muhlert et al., 2010; Wilkinson et al., 2012). Such correlations can provide insight into whether ALF is associated with an early or a later memory deficit. We used Pearson correlations to examine the relationship between “NART-predicted verbal IQ” and 1-week retention scores. ANCOVAs with covariate NART-predicted verbal IQ were run to examine whether the reported memory findings persisted when controlling for the subtle group difference in NART-predicted verbal IQ.

The Greenhouse–Geisser correction for nonsphericity was applied if the sphericity assumption (according to the Mauchly’s test of sphericity) was violated. Effect sizes for the ANOVAs were determined using partial η^2^, where 0.14 is a large effect (Stevens, 2002). The α level was set to 0.05 for all analyses.

## Results

### Word-list learning

The TEA patients acquired the lists to a similar level as the controls: there was a significant main effect of learning trial, *F*(1, 25) = 118.587, *p* < 0.001, η_p_^2^ = .826, but no significant main effect of group, *F*(1, 25) = 1.361, *p* = 0.254, η_p_^2^ = .052, or a significant Group × Learning trial interaction, *F*(1, 25) = 0.956, *p* = 0.337, η_p_^2^ = .037). Immediate recall performance improved from learning Trial 1 (*M*_Patients_ = 7.05 ± 1.88 words, *M*_Controls_ = 7.94 ± 2.39 words) to learning Trial 2 (*M*_Patients_ = 9.07 ± 2.21 words, *M*_Controls_ = 10.36 ± 2.93 words) at similar rates for patients and controls.

### One-Week Performance

**Figure 2 fig2:**
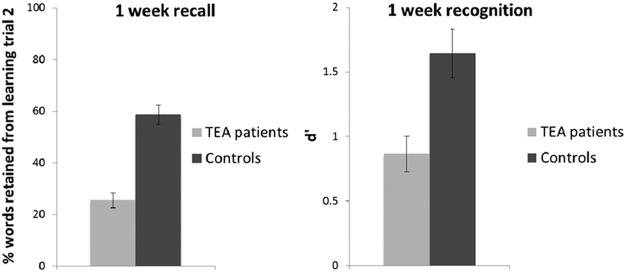
One-week delayed recall and recognition performance. Recall test: mean percentage of words retained from learning Trial 2 after 1 week by transient epileptic amnesia (TEA) patients (light grey) and controls (dark grey). Recognition test: mean *d*’ score after 1 week. Error bars represent the standard error of the mean (*SEM*).

Figure 2 shows average percentage word-list retention and word recognition 1 week after word-list learning. Percentage retention after 1 week was significantly lower in the TEA patients than in the controls, *t*(25) = −6.357, *p* < .001, *r* = .79. Moreover, performance on the 1-week recognition test, that is, *d*′ score, was also significantly lower in the TEA patients than in the controls, *t*(25) = −3.087, *p* < .01, *r* = .53 (see online Supplementary Data 2 for hit and false-alarm rates). The number of words recalled after 1 week (i.e., absolute scores) correlated significantly with the 1-week word-recognition performance in the TEA patients (*r =* .608, *p* < .05) and in the controls (*r* = .676, *p* < .01).[Fig-anchor fig2]

### Delayed Recall Performance Within the First 24 Hours

**Figure 3 fig3:**
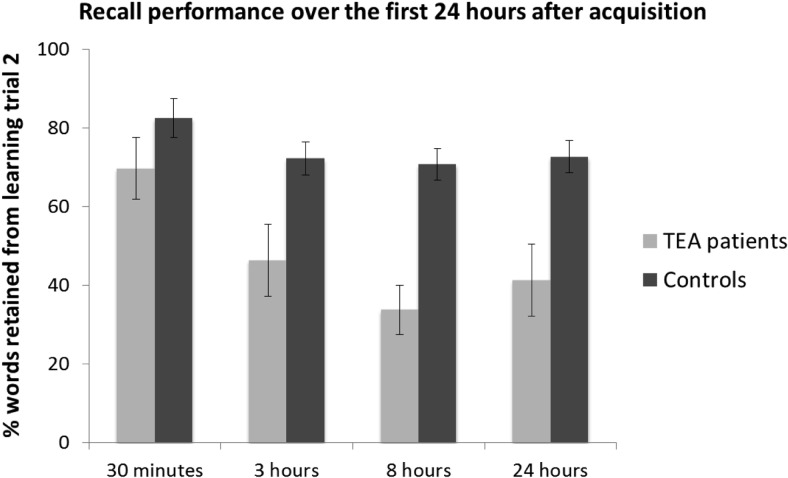
Recall performance over the first 24 hr after acquisition. Mean percentage of words retained from learning Trial 2 after 30 min, 3 hr, 8 hr, and 24 hr by transient epileptic amnesia (TEA) patients (light grey) and healthy control participants (dark grey). Error bars represent the standard error of the mean (*SEM*).

Figure 3 shows percentage retention scores over the first 24 hr in the TEA and control groups.[Fig-anchor fig3]

The analysis of percentage of words retained (from the number of words recalled at learning Trial 2) across the four delay intervals within the first 24 hr after acquisition (i.e., 30 min, 3, 8 and 24 hr) revealed a significant main effect of delay, *F*(3, 75) = 7.005, *p* < 0.001, η_p_^2^ = .219, and a significant main effect of group, *F*(1, 25) = 27.071, *p* < .001, η_p_^2^ = .520, but no significant interaction between group and delay, *F*(3, 75) = 1.744, *p* = .165, η_p_^2^ = .065.

As shown in Figure 3, the patients’ retention scores dropped over the 30-min to 3-hr interval, and over the 3-hr to 8-hr interval. Although these consecutive drops in patients’ retention were not significant, *t*(10) = 1.950, *p* = .080, *r* = .52; *t*(10) = 1.378, *p* = .198, *r* = .40, respectively, the cumulative drop in the patients’ retention over the 30-min to 8-hr interval was significant, *t*(10) = 3.296, *p* < .01, *r* = .72. This was also the case for the cumulative drop in the patients’ retention over the 30-min to 24-hr interval, *t*(10) = 2.786, *p* < .05, *r* = .66, though no significant further drop in retention was observed over the 8-hr to 24-hr interval, *t*(10) = −0.693, *p* = .504, *r* = .21, or cumulatively over the 3-hr to 24-hr interval, *t*(10) = 0.465, *p* = .652, *r* = .15. In fact, Figure 3 shows that there was a subtle, nonsignificant increase in retention over the 8-hr to 24-hr interval in the patients.

The controls’ retention scores dropped over the 30-min to 3-hr interval, but this drop was not significant, *t*(15) = 1.824, *p* = .088, *r* = .43. Moreover, their retention did not drop significantly over the subsequent 3-hr to 8-hr interval, *t*(15) = 0.248, *p* = .808, *r* = .06. However, the cumulative drop in the controls’ retention over the 30-min to 8-hr interval approached significance, *t*(15) = 2.121, *p* = .051, *r* = .48. This was not the case for the cumulative drop in controls’ retention over the 30-min to 24-hr interval, *t*(15) = 1.657, *p* = .118, *r* = .39. Their retention did not drop further over the 8-hr to 24-hr interval, *t*(15) = - 0.402, *p* = .693, *r* = .10.

Figure 3 also shows that, whereas the patients’ and controls’ percentage retention scores did not differ significantly after 30 min, *t*(25) = −1.447, *p* = .160, *r* = .28, the patients’ percentage retention scores were significantly lower than those of the controls after 3 hr, *t*(14.189) = −2.561, *p* < .05, *r* = .56, after 8 hr, *t*(25) = −5.262, *p* < .001, *r* = .72, and after 24 hr, *t*(14.031) = −3.144, *p* < .01, *r* = .64. Indeed, retention dropped significantly more in the patients than in the controls over the 30-min to 8-hr interval: Group × Delay interaction, *F*(1, 25) = 4.678, *p* < .05, η_p_^2^ = .158. This was not the case for the drop in retention over the 30-min to 3-hr interval: no significant Group × Delay interaction, *F*(1, 25) = 1.193, *p* = .285, η_p_^2^ = 0.046, or for the 30-min to 24-hr interval: no significant Group × Delay interaction, *F*(1, 25) = 2.813, *p* = .106, η_p_^2^ = .101.

### Delayed Recall Performance Within the First 24 Hours: Incorrect Responses and Guess Corrections

The number of incorrect responses across the 24-hr interval was low in both groups (30-min mean: TEA = 0.82, controls = 0.56; 3-hr mean: TEA = 0.64, controls = 0.81, 8-hr mean: TEA = 1.64, controls = 0.56; 24-hr mean: TEA = 1.27, controls = 0.5) and did not increase significantly over the course of the 24-hr period, *F*(3, 75) = 1.406, *p* = .248, η_p_^2^ = 0.053. The TEA patients produced more incorrect responses than did the controls at the 8-hr test and 24-hr test. Whereas the former group difference was nonsignificant, *t*(14.287) = 1.647, *p* = .121, *r* = .40, the latter was near-significant, *t*(25) = 2.006, *p* = .056, *r* = .37. However, the number of incorrect responses did not increase significantly from the 8-hr to the 24-hr test in either the TEA patients, *t*(10) = 0.714, *p* = .492, *r* = .22 or the controls, *t*(15) = 0.293, *p* = .774, *r* = .08.

Correction for potential guessing (see Materials and Method for guess-correction method) did not change the results for retention over the first 24 hr for either patients or controls, except that the drop in the controls from 30-min to 3-hr, and from 30-min to 8-hr became statistically significant (*p* < .05). The percentage retention scores still differed significantly between groups after 3 hr, *t*(16.355) = −2.262, *p* < .05, *r* = .49, after 8 hr, *t*(25) = −4.382, *p* < .001, *r* = .66, and after 24 hr, *t*(14.583) = −3.585, *p* < .01, *r* = .68. Moreover, the significant Group × Delay interaction over the 30-min to 8-hr interval, *F*(1, 25) = 4.513, *p* < .05, η_p_^2^ = .153 persisted. Thus, retention dropped significantly more in the patients than the controls over this time interval, even after correction for potential guessing. The patients’ mean guess-corrected retention scores (and *SEM*s) were 65.53% (8.69) at the 30-min interval, 43.64% (9.13) at the 3-hr interval, 29.29% (6.89) at the 8-hr interval, and 34.88% (8.56) at the 24-hr interval. The controls’ mean guess-corrected retention scores were 79.64% (5.87) at the 30-min interval, 67.38% (5.18) at the 3-hr interval, 66.53% (5.22) at the 8-hr interval, and 68.9% (4.09) at the 24-hr interval. All guess-corrected analyses are reported in online Supplementary Data 3.

### Correlations Between Early Forgetting and Late Forgetting

#### Word list recalled after 30 min and after 1 week

Early forgetting (final learning trial to 30 min) did not correlate significantly with late forgetting (30 min to 1 week) in the patients (*r* = −.240, *p* = .477, *n* = 11) or the controls (*r* = .388, *p* = .138, *n* = 16).

#### Word list recalled after 3 hr and after 1 week

Early forgetting (final learning trial to 3 hr) did not correlate significantly with late forgetting (3 hr to 1 week) in the patients (*r* = −.195, *p* = .614, *n* = 9) or the controls (*r* = −.112, *p* = .681, *n* = 16).

#### Word list recalled after 8 hr and after 1 week

Early forgetting (final learning trial to 8 hr) did not correlate significantly with late forgetting (8 hr to 1 week) in the patients (*r* = −.584, *p* = .098, *n* = 9) or the controls (*r* = −.145, *p* = .593, *n* = 16).

#### Word list recalled after 24 hr and after 1 week

Early forgetting (final learning trial to 24 hr) did not correlate significantly with late forgetting (24 hr to 1 week) in the patients (*r* = −.530, *p* = .142, *n* = 9) or the controls (*r* = .067, *p* = .805, *n* = 16).

It should be noted that one patient scored 0 at the 3-, 8- and 24-hr intervals, and three patients had a score of 0 at one of these three intervals. These patients were excluded from the corresponding correlations, as no forgetting could be measured over the corresponding late-forgetting interval (the 3-hr to 1-week, 8-hr to 1-week and 24-hr to 1-week intervals).

### IQ Scores and ALF

As indicated in the Materials and Method section, the control group outperformed the patient group in the NART, resulting in a subtle, albeit significant group difference in NART-predicted verbal IQ, *t*(25) = −2.076, *p* = .048, *r* = .38 (see Table 2). However, the 1-week word-list retention scores did not correlate significantly with the NART-predicted verbal IQ levels among either the TEA patients (r = −.230, *p* = .496) or the controls (*r* = .364, *p* = .166). Moreover, inclusion of the NART-predicted verbal IQ as a covariate in the analysis had minimal effect on our main findings: The percentage retention scores remained significantly different between the TEA patients and the controls after 8 hr, 24 hr and 1 week, and the group difference was close to significance after 3 hr: 30 min, *F*(1, 24) = 0.530, *p* = .473, η_p_^2^ = .022; 3 hr, *F*(1, 24) = 3.997, *p* = .057, η_p_^2^ = .143; 8 hr, *F*(1, 24) = 19.279, *p* < .001, η_p_^2^ = .445; 24 hr, *F*(1, 24) = 10.043, *p* < .01, η_p_^2^ = .295; 1 week, *F*(1, 24) = 30.718, *p* < .001, η_p_^2^ = .561. The significant Group × Delay interaction over the 30-min to 8-hr interval also persisted after controlling for the subtle group difference in NART-predicted verbal IQ, *F*(1, 24) = 4.293, *p* < .05, η_p_^2^ = .152. ANCOVA results for the guess-corrected data were similar (see online Supplementary Data 4 for all ANCOVA analyses with NART-predicted verbal IQ as covariate).

## Discussion

There is compelling evidence that accelerated long-term forgetting occurs in some patients with TLE, and especially TEA, in whom memory tests give normal results at standard delays (Butler & Zeman, 2008; Fitzgerald, Thayer, Mohamed, & Miller, 2013; Mameniskiene, Jatuzis, Kaubrys, & Budrys, 2006; Narayanan et al., 2012; Wilkinson et al., 2012; Zeman et al., 2013). The aim of the present study was to examine whether ALF becomes apparent during the day of memory acquisition, and, if so, when it becomes apparent. Our key finding is that ALF can be detected within 3–8 hr of learning among patients showing ALF at one week, without any requirement for intervening sleep. Whereas patients scored comparably to controls immediately after the second learning trial, and after 30 min, they retained significantly fewer words than did the control group at the 3-hr, 8-hr and 24-hr junctures. Of importance, the TEA group’s retention dropped significantly more than did the controls’ from 30 min to 8 hr, thus demonstrating ALF over the first 8 hr postacquisition. No further forgetting was observed over the first night in either group (i.e., between 8 hr and 24 hr; see Figure 3). In fact, the patients’ retention scores increased slightly (though not significantly) from the 8-hr to the 24-hr delay interval.

The high mean IQ of our patient group is of interest. This has been a consistent finding in research on TEA (Zeman & Butler, 2010). It may be the result of an ascertainment bias, reflecting the difficulty of diagnosing this underrecognized disorder in less articulate individuals who make less intensive demands of memory. It is possible, alternatively, that the prevalence of this disorder is genuinely increased among individuals of higher IQ.

The TEA patients’ mean NART-predicted verbal IQ was slightly lower than that of the controls. However, this small group difference is unlikely to be relevant to the pattern of ALF that we have observed among patients. First, retention scores observed after 1 week were not correlated significantly with NART-predicted verbal IQ levels among either the TEA patients or the controls. Second, the accelerated memory loss in the TEA patients over the 30-min to 8-hr interval was not affected by controlling for the group difference in NART-predicted verbal IQ. Finally, retention scores differed significantly between groups at the 8-hr, 24-hr, and 1-week test intervals, even when controlling for the small difference in IQ (see online Supplementary Data 4 for all analyses).

What is the cognitive basis of the emergence of accelerated forgetting at 3–8 hr after learning? It is unlikely that it can be accounted for primarily by an acquisition deficit. The use of a cued category test was expected to reduce the need for elaborate encoding strategies as each list contained items from a specific category (e.g., animals), and was preceded by the category title, providing a semantic framework for encoding. The TEA patients learned as rapidly as controls, and benefitted as much as the controls from a second learning trial, at least in terms of total words recalled. Nonetheless, they showed significantly more forgetting over the first 8 hr than did the controls. This performance pattern is in keeping with previous findings in TEA which argue against an acquisition-deficit hypothesis (e.g., Butler et al., 2007; Hoefeijzers et al., 2013; Muhlert et al., 2010; Manes et al., 2005). We acknowledge that the equivalent number of words retrieved at immediate recall in TEA and controls does not necessarily imply equivalence in memory *strength,* which cannot be measured fully via tests of verbal recall (Kornell, Bjork, & Garcia, 2011). A subtle reduction of memory strength at the time of memory formation could account for the small but significant impairment of recall at 30 min noted by Butler et al. (2007). It could also account for related findings in patients with TLE, such as the impaired word-pair recall observed by McGibbon and Jansari (2013) at an interval of 30 min in a single case study, and the impaired story recall reported by Wilkinson et al. (2012) at 1 hr.

The excellent performance of some patients at 30 min in the present study provides prima facie evidence against an interpretation of ALF in terms of reduced memory strength at acquisition: seven of the 11 TEA patients had 30-min retention scores within 1 *SD* of the control mean (62.57–100% retention). Moreover, three of these patients retained 100% after 30 min. In keeping with these experimental test findings, 30-min retention of the WMS-III (Wechsler, 1997) logical memory story did not differ significantly between the TEA patients and controls (see Table 1). In fact, nine of the 11 TEA patients (81.82%), and 14 of the 16 controls (87.5%) retained 80% or more of this story after the 30-min delay. This notwithstanding, further work, using both neuropsychological and neurobiological methods will be required to rule out an acquisition hypothesis of ALF in TEA.

It is also unlikely that a retrieval deficit can account for the ALF demonstrated here. The use of cued recall and category lists facilitates retrieval, and should reduce any subtle retrieval deficits in the patient group. Indeed patients’ recall was unimpaired at 30 min, but patients performed significantly more poorly than controls at word-list recall at all subsequent delays. Moreover, the use of a word-recognition test at 1 week, which further facilitates retrieval, failed to normalize performance in the patient group.

Instead, the present finding supports the view that ALF is associated with an impairment of consolidation. Previous research has provided evidence for the hypothesis that ALF for verbal material in TEA reflects an impairment of memory consolidation that takes place over the first 24 hr (Zeman & Butler, 2010; see also Jansari et al., 2010; Muhlert et al., 2010). The present finding goes further, supporting the hypothesis that ALF in TEA reflects an impairment of memory consolidation over the first few hr after acquisition, rather than an impairment in sleep-related consolidation processes. Indeed, there was a slight, nonsignificant, improvement in retention over the first night in the patients (i.e., within the 8–24-hr delay interval; see Figure 3).

The demonstration of ALF after 3–8 hr of wakefulness following new learning shows that disturbance of sleep related memory processes is *not necessary* for ALF to emerge in TEA. Whether or not *wakefulness* is necessary for ALF to emerge on the other hand cannot be inferred directly from the present paradigm. Since learning was always followed immediately by wakefulness, and never by sleep, it is possible that in patients showing ALF after 1 week, ALF becomes apparent 3–8 hr after learning, irrespective of behavioral state, that is, sleep/wakefulness. However, a recent study on ALF in TLE patients speaks against this ‘nonspecific’ early forgetting hypothesis. Deak et al. (2011) observed ALF in TLE when learning was followed by a 12-hr period of daytime wakefulness, but not when learning was followed by a 12-hr period of nocturnal sleep. In keeping with these results, Atherton, Nobre, Zeman, and Butler (2014) have very recently reported the occurrence of ALF over a period of 12 hr of wakefulness, but not over a comparable period of sleep in a group of patients with TEA. These finding suggests that ALF in TEA is typically associated with memory-consolidation deficits occurring during wakefulness specifically.

This daytime consolidation deficit could be associated with memory interference, produced by novel encoding. Indeed, the encoding of novel material interferes mildly with early consolidation in healthy people (Dewar, Alber et al., 2012) and severely so in patients with anterograde amnesia (Cowan et al., 2004; Dewar et al., 2009, 2010; Dewar, Pesallaccia et al., 2012). Alternatively, although all patients were on antiepileptic drug treatment and free from overt seizures for the last 6 months, subclinical epileptiform activity could also interfere with the daytime consolidation processes of our TEA patients (e.g., Butler & Zeman, 2008; Evans, Elliott, Reynders & Isaac, 2014; Fitzgerald et al., 2013; Gascoigne, Barton, Webster, Gill, Antony & Lah, 2012).

If, as our findings suggest, ALF can be associated with an impairment of daytime consolidation, what is the underlying mechanism? Determining the precise nature and timing of the consolidation deficit associated with ALF as revealed in our study requires further research, probing neurobiological as well as behavioral processes. It may well be that a range of successive stages of memory processing can be involved. This hypothesis is biologically plausible (e.g., Lamprecht, & LeDoux, 2004; Reymann & Frey, 2007) and in keeping with the observed absence of significant correlations between early- and late-forgetting rates. This hypothesis could help to account for the apparent heterogeneity of ALF, evidenced by the five patients we excluded from this study due to normal performance on our experimental test. These patients reported ALF over days or weeks rather than hr, consistent with an explanation of their complaint in terms of “slow ALF” due to impairment of late consolidation processes. It is possible also, however, that these patients have a similar consolidation deficit to patients showing ALF at shorter intervals, but of milder degree. This might not have been captured by our experimental test which minimized encoding and retrieval demands (for a review of the methodology of ALF testing, see Elliott, Isaac & Muhlert, 2014). Finally, it could be that some reports of ALF reflect factors unrelated to basic memory processes, such as elevated expectations of performance or lowered mood. The heterogeneity of ALF requires further exploration.

## Supplementary Material

10.1037/neu0000114.supp

## Figures and Tables

**Table 1 tbl1:** Transient Epileptic Amnesia (TEA) Patient Information

Gender	Age	Age at onset	Evidence for a diagnosis of epilepsy			Imaging
			EEG	Other features	Treatment response	
M	69	67	Not performed	none	Complete	Normal CT
M	66	61	Normal	none	Complete	Normal MRI
M	66	55	Normal	none	Complete	Normal MRI
M	71	65	Normal	TCL	Complete	Normal MRI
M	75	67	Normal	SPS, CPS	Complete	Normal MRI
M	76	73	Normal	CPS	Complete	Abnormal MRI*
M	67	66	Epileptiform (L)	none	Complete	Normal MRI
M	73	71	Epileptiform (L)	SPS	Complete	Normal MRI
M	74	67	Epileptiform (L)	CPS	Complete	Normal MRI
M	74	53	Epileptiform (BL)	SPS	Partial	Normal MRI
F	57	Mid-20s	Epileptiform (BL)	TCL, CPS	Partial	Abnormal MRI**
*Note.* SPS = simple partial seizures; CPS = complex partial seizures; TCL = tonic–clonic seizures. (L) = left; (BL) = bilateral epileptiform. EEGs were compatible with a temporal lobe origin.						
* Small area of right frontal encephalomalacia and slightly excessive global atrophy on MRI. ** Left hippocampal cyst; left putaminal T2 hypointensity.						

**Table 2 tbl2:** Demographic, Clinical and Neuropsychological Profile of Transient Epileptic Amnesia (TEA) Patients Showing ALF After One Week and Control Participants

Variable	TEA patients (*n* = 11)	Controls (*n* = 16)
	Mean (*SD*)	Mean (*SD*)
Age (years)	69.82 (5.60)	67.00 (4.05)
Sex distribution	10 M, 1 F	8 M, 8 F
Education (years)	12.73 (2.97)	14.88 (3.54)
IQ measures (max score)		
NART-predicted verbal IQ (129)	116.87 (7.37)	121.99 (5.45) *
WASI similarities test–scaled scores (19)	12.64 (1.63)	13.63 (1.54)
WASI matrix reasoning–scaled scores (19)	13.55 (2.62)	14.06 (1.84)
Episodic memory scores (max score)		
WMS-III Logical memory immediate recall–raw scores (25)	14.73 (3.77)	16.38 (2.83)
WMS-III Logical memory delayed recall–raw scores (25)	13.00 (4.69)	15.88 (3.26)
WMS-III Logical memory % retention (100)	85.80 (15.22)	94.36 (11.14)
WMS-III Logical memory recognition test–raw scores (15)	12.91 (1.51)	13.56 (1.36)
Rey figure delayed recall–raw scores (36)	20.23 (5.23)	21.84 (6.55)
Visuospatial perception (max score)		
Rey figure copy–raw scores (36)	34.82 (2.14)	35.31 (1.20)
Executive function		
FAS Letter fluency–raw scores (words/3 min)	37.09 (8.22)	44.94 (12.79)
Mood score (max score)		
HADS (42)	10.73 (4.94)	6.94 (3.73) *
*Note.* ALF = accelerated long-term forgetting; NART = National Adult Reading Test; WASI = Wechsler Abbreviated Scale of Intelligence; WMS-III = Wechsler Memory Scale-III; HADS = Hospital Anxiety and Depression Scale. WMS-III Logical memory % retention = (delayed recall/immediate recall) × 100; numbers in parentheses after test name = maximum score on that test.		
* *p* < 0.05.
