# Supplementary material for: Accelerated Long-Term Forgetting Can Become Apparent Within 3–8 Hours of Wakefulness in Patients With Transient Epileptic Amnesia
Source: Neuropsychology. 2014 Aug 4;29(1):117–25. doi: 10.1037/neu0000114 (PMC4296931; doi:10.1037/neu0000114)
Supplement: Supplementary file 1 [file nps-NEU-2013-1324-Supplementary_Data_1.docx]

| **Supplementary Data 1**  Immediate recall performance after learning trial 1 and 2 on four categorical word lists titled “Animals”, “City”, “Nature” and “Groceries”. | | | |
| --- | --- | --- | --- |
| **Categorical word list** | **TEA Patients** |  | **Controls** |
|  | **Mean (SD)** |  | **Mean (SD)** |
| *First learning trial* |  |  |  |
| Animals | 6.82 (1.40) |  | 8.19 (2.99) |
| City | 7.64 (2.62) |  | 7.44 (2.83) |
| Groceries | 6.82 (2.48) |  | 7.69 (3.09) |
| Nature | 6.91 (2.51) |  | 8.44 (1.71) |
| *Second learning trial* |  |  |  |
| Animals | 9.00 (2.10) |  | 10.50 (3.58) |
| City | 9.64 (2.42) |  | 10.44 (2.71) |
| Groceries | 8.45 (2.77) |  | 10.38 (2.80) |
| Nature | 9.18 (2.52) |  | 10.13 (3.20) |
| A mixed factors ANOVA with within factors ‘learning trial’ (learning trial 1 vs. learning trial 2) and ‘word list’ (Animals vs. City vs. Nature vs. Groceries) and between factor ‘group’ (TEA patients versus Controls) revealed:  A significant main effect of learning trial (*F*(1,25) = 118.587, *p* < 0.001, η^2^_p_= .826). However, this effect of learning trial did not differ between patients and controls (no significant learning trial * group interaction: *F*(1,25) = 0.956, *p* = 0.337, η^2^_p_= .037).  No significant main effect of word list (*F*(3,75) = 0.917, *p* = 0.437, η^2^_p_= .035). Moreover, word list recall performance did not differ between patients and controls (no significant word list * group interaction: *F*(3,75) = 1.775, *p* = 0.159, η^2^_p_= .066).  Recall performance over the 2 learning trials did not differ between word lists (no significant word list * learning trial interaction: *F*(3,75) = 0.387, *p* = 0.763, η^2^_p_= .015). Moreover, recall performance over the 2 learning trials for all word lists was similar between patients and controls (no significant word list * learning trial * group interaction: *F*(3,75) = 1.261, *p* = 0.294, η^2^_p_= .048). | | | |
