# Supplementary material for: Accelerated Long-Term Forgetting Can Become Apparent Within 3–8 Hours of Wakefulness in Patients With Transient Epileptic Amnesia
Source: Neuropsychology. 2014 Aug 4;29(1):117–25. doi: 10.1037/neu0000114 (PMC4296931; doi:10.1037/neu0000114)
Supplement: Supplementary file 2 [file nps-NEU-2013-1324-Supplementary_Data_2.docx]

| **Supplementary Data 2: Word recognition test (1-week after word list learning)**  Recognition performance of TEA patients and controls: hit rates for originally presented words (i.e. target items) and false alarm (FA) rates for all foils, as well separately for *semantically distant – phonetically related* foils, *semantically close – phonetically unrelated* foils, *semantically distant – phonetically unrelated* foils. | | | |
| --- | --- | --- | --- |
|  | **TEA patients**  Mean (St. Error) | **Controls**  Mean (St. Error) |  |
| Hit rate (out of 64 targets)  False alarm rate of all foils combined (out of 64) | 0.67 (0.04)  0.37 (0.05) | 0.79 (0.03)  0.26 (0.04) | *p* < .05  *p* = .080 |
| False alarm rate of *semantically distant – phonetically related* foils (out of 24) | 0.25 (0.04) | 0.19 (0.04) | *p* = .306 |
| False alarm rate of *semantically close – phonetically unrelated* foils (out of 20) | 0.39 (0.07) | 0.26 (0.05) | *p* = .116 |
| False alarm rate of *semantically distant – phonetically unrelated* foils (out of 20) | 0.50 (0.07) | 0.33 (0.06) | *p* = .059 |
|  |  |  |  |
| ***Note:*** the 16 original words *(i.e. the targets)* of each list were intermixed with 16 foils. Because the test consisted of 4 lists, the recognition tests consisted of 64 target items and 64 foils.  Per list:   - 6 foils were *semantically distant – phonetically related* (e.g. ‘mouse’ and ‘moose’) (4x 6 = 24 in total) - 5 foils were *semantically close – phonetically unrelated* (e.g. ‘lion’ and ‘tiger’) (4x 5 = 20 in total) - 5 foils were *semantically distant – phonetically unrelated* (e.g. ‘snake’ and ‘bear’) (4x 5 = 20 in total)   *p*-values represent One-way ANOVA analyses between groups (TEA patients vs. controls) | | | |
