# Supplementary material for: Accelerated Long-Term Forgetting Can Become Apparent Within 3–8 Hours of Wakefulness in Patients With Transient Epileptic Amnesia
Source: Neuropsychology. 2014 Aug 4;29(1):117–25. doi: 10.1037/neu0000114 (PMC4296931; doi:10.1037/neu0000114)
Supplement: Supplementary file 3 [file nps-NEU-2013-1324-Supplementary_Data_3.docx]

**Supplementary Data 3: All results using the guess-corrected data**

**Wordlist learning**

The TEA patients acquired the lists to a similar level as the controls: there was a significant main effect of learning trial (*F*(1,25) = 116.962, *p <* 0.001, η^2^_p_= .824), but no significant main effect of group (*F*(1,25) = 1.940, *p =* 0.176, η^2^_p_= .072) or a significant group x learning trial interaction (*F*(1,25) = 0.969, *p =* 0.334, η^2^_p_= .037). Immediate guess-corrected recall performance improved from learning trial 1 (*M*_Patients_ = 6.68±1.97 words, *M*_Controls_ = 7.77±2.31 words) to learning trial 2 (*M*_Patients_ = 8.75±2.15 words, *M*_Controls_ = 10.25±2.95 words) at similar rates for patients and controls.

**1-week performance**

The patients’ and controls’ mean guess-corrected retention scores (and SEMs) at the 1-week interval were respectively 17.83% (10.22) and 51.50% (16.31). Guess-corrected percentage retention after 1 week was significantly lower in the TEA patients than in the controls (*t*(25)= -6.055, *p* < 0.001, *r* = .77). The guess-corrected number of words recalled after 1 week (i.e. absolute scores) correlated significantly with the 1-week word recognition performance in the TEA patients (*r =* .669, *p* < .05) and in the controls (*r* = .565, *p* < 0.05).

**Delayed recall performance within the first 24 hours**

The patients’ mean guess-corrected retention scores (and SEMs) were 65.53% (8.69) at the 30-min interval, 43.64% (9.13) at the 3-hour interval, 29.29% (6.89) at the 8-hour interval, and 34.88% (8.56) at the 24-hour interval. The controls’ mean guess-corrected retention scores were 79.64% (5.87) at the 30-min interval, 67.38% (5.18) at the 3-hour interval, 66.53% (5.22) at the 8-hour interval, and 68.9% (4.09) at the 24-hour interval.

The analysis of guess-corrected percentage of words retained (from the number of words recalled at learning trial 2) across the four delay intervals within the first 24 hours after acquisition (i.e. 30 minutes, 3, 8 and 24 hours) revealed a significant main effect of delay (*F*(3,75) = 7.756, *p <* 0.001, η^2^_p_= .237) and a significant main effect of group (*F*(1,25) = 18.449, *p* < 0.001, η^2^_p_= .425), but no significant interaction between group and delay (*F*(3, 75) = 1.812, *p* = 0.152, η^2^_p_= .068). The patients’ guess-corrected retention scores dropped over the 30-min to 3-hour interval, and over the 3-hour to 8-hour interval. While these consecutive drops in the patients’ guess-corrected retention were not significant (*t*(10) = 1.945, *p* = 0.080, *r* = .52; *t*(10) = 1.473, *p* = 0.171, *r* = .42, respectively), the cumulative drop in the patients’ guess-corrected retention over the 30-min to 8-hour interval was significant (*t*(10) = 3.457, *p* < 0.01, *r* = .74). This was also the case for the cumulative drop in the patients guess-corrected retention over the 30-min to 24-hour interval (*t*(10) = 3.458, *p* < 0.01, *r* = .74), though no significant further drop in retention was observed over the 8-hour to 24-hour interval (*t*(10) = -0.508, *p* = 0.623, *r* = .16), or cumulatively over the 3-hour to 24-hour interval (*t*(10) = 0.807, *p* = 0.438, *r* = .25). In fact, there was a subtle, non-significant increase in retention over the 8-hour to 24-hour interval in the patients (i.e. from 29.29% to 34.88%).

The controls’ guess-corrected retention scores dropped significantly over the 30-min to 3-hour interval (*t*(15) = 2.145, *p* < 0.05, *r* = .48). Their guess-corrected retention did not drop significantly over the subsequent 3-hour to 8-hour interval (*t*(15) = 0.135, *p* = 0.895, *r* = .03). The cumulative drop in the controls’ guess-corrected retention over the 30-min to 8-hour interval was significant (*t*(15) = 2.388, *p* < 0.05, *r* = .52). This was not the case for the cumulative drop in the controls’ guess-corrected retention over the 30-min to 24-hour interval (*t*(15) = 1.675, *p* = 0.115, *r* = .39). Their guess-corrected retention did not drop further over the 8-hour to 24-hour interval (*t*(15) = - 0.508, *p* = 0.619, *r* = .13).

Whereas the patients’ and controls’ guess-corrected percentage retention scores did not differ significantly after 30 minutes (*t*(25)= -1.398, *p* = 0.174, *r* = .27), the patients’ guess-corrected percentage retention scores were significantly lower than those of the controls after 3 hours (*t*(16.355)= -2.262, *p* < 0.05, *r* = .49), after 8 hours (*t*(25)= -4.382, *p* < 0.001, *r* = .66) and after 24 hours (*t*(14.583)= -3.585, *p* < 0.01, *r* = .68). Indeed, guess-corrected retention dropped significantly more in the patients than the controls over the 30-minute to 8-hour interval (group x delay interaction, *F*(1,25) = 4.513, *p* < 0.05, η^2^_p_= .153). This was not the case for the drop in guess-corrected retention over the 30-minute to 3-hour interval (no significant group x delay interaction, *F*(1,25) = 0.695, *p* = 0.412, η^2^_p_= 0.027), or for the 30-minute to 24-hour interval (no significant group x delay interaction, *F*(1,25) = 3.493, *p* = 0.073, η^2^_p_= .123).

**Correlations between early forgetting and late forgetting**

**Word list recalled after 30 minutes and after 1 week.** Early forgetting (final learning trial to 30 minutes) did not correlate significantly with late forgetting (30 minutes to 1 week) in the patients (*r* = -.315, *p* = 0.345, n=11), but there was a significant correlation in the control group (*r* = .539, *p* < 0.05, n=16).

**Word list recalled after 3 hours and after 1 week.** Early forgetting (final learning trial to 3 hours) did not correlate significantly with late forgetting (3 hours to 1 week) in either the patients (*r* = .113, *p* = 0.772, n= 9) or the controls (*r* = -.226, *p* = 0.399, n=16).

**Word list recalled after 8 hours and after 1 week.** Early forgetting (final learning trial to 8 hours) did not correlate significantly with late forgetting (8 hours to 1 week) in either the patients (*r* = -.417, *p* = 0.264, n= 9) or the controls (*r* = -.128, *p* = 0.637, n=16).

**Word list recalled after 24 hours and after 1 week.** Early forgetting (final learning trial to 24 hours) did not correlate significantly with late forgetting (24 hours to 1 week) in either the patients (*r* = -.452, *p* = 0.221, n= 9) or the controls (*r* = .093, *p* = 0.733, n= 16).

It should be noted that one patient scored zero at the 3-, 8- and 24-hour intervals, while three patients had a zero score at one of these three intervals. These patients were excluded from the corresponding correlations as no forgetting could be measured over the corresponding late forgetting interval (the 3-hour to 1-week, 8-hour to 1-week and 24-hour to 1-week interval).

**IQ scores and ALF**

No correlation was found between 1-week retention scores and NART- IQ levels for either the TEA patients (r = -.183, *p* =.591) or the controls (r =.421, *p* =.104) . Also for the guess corrected data, no correlation was found between 1-week retention scores and NART-predicted verbal IQ levels for either the TEA patients (r = -.183, p =.591) or the controls (r =.421, p =.104). Moreover, when the data were corrected for potential guessing (see Materials and Methods for method) and controlled for the subtle verbal IQ difference between groups, no substantial changes were observed in the overall findings. That is, the retention scores remained significantly different between the TEA patients and the controls after 8 hours, 24 hours and one week (30 minutes: F(1,24) = 0.386, p = 0.540, η^2^_p_= .016; 3 hours: F(1,24) = 2.487, p = 0.128, η^2^_p_= .094; 8 hours: F(1,24) = 12.752, p < 0.01, η^2^_p_= .347; 24 hours: F(1,24) = 13.001, p = 0.001, η^2^_p_= .351; 1 week: *F*(1,24) = 27.220, *p* < 0.001, η^2^_p_= .531). The group x delay interaction over the 30-minute to 8-hour interval also remained significant (F(1,24) = 4.583, p < 0.05, η^2^_p_= .160).

*See Supplementary Data 4 for all analyses where the guess corrected data are controlled for the subtle group differences in “NART-predicted verbal IQ”.*
