# Supplementary material for: Accelerated Long-Term Forgetting Can Become Apparent Within 3–8 Hours of Wakefulness in Patients With Transient Epileptic Amnesia
Source: Neuropsychology. 2014 Aug 4;29(1):117–25. doi: 10.1037/neu0000114 (PMC4296931; doi:10.1037/neu0000114)
Supplement: Supplementary file 4 [file nps-NEU-2013-1324-Supplementary_Data_4.docx]

| **Supplementary Data 4**  Group differences in wordlist retention after controlling for subtle group differences in NART-predicted verbal IQ - **ANCOVAs** (analysis of covariance) using “NART-predicted verbal IQ” as covariate | | | | | |
| --- | --- | --- | --- | --- | --- |
|  |  | **Uncorrected data** |  | **Guess-corrected data** |  |
|  |  | *TEA patients vs. controls* |  | *TEA patients vs. controls* |  |
| *Learning trial 1* |  | *F*(1,24) = 0.074, *p* = 0.788, η^2^_p_= .003 |  | *F*(1,24) = 0.268, *p* = 0.609, η^2^_p_= .011 |  |
| *Learning trial 2* |  | *F*(1,24) = 0.220, *p* = 0.643, η^2^_p_= .009 |  | *F*(1,24) = 0.468, *p* = 0.501, η^2^_p_= .019 |  |
| *30 minutes* |  | *F*(1,24) = 0.530, *p* = 0.473, η^2^_p_= .022 |  | *F*(1,24) = 0.386, *p* = 0.540, η^2^_p_= .016 |  |
| *3 hours* |  | *F*(1,24) = 3.997, *p* = 0.057, η^2^_p_= .143 |  | *F*(1,24) = 2.487, *p* = 0.128, η^2^_p_= .094 |  |
| *8 hours* |  | *F*(1,24) = 19.279, *p* < 0.001, η^2^_p_= .445 |  | *F*(1,24) = 12.752, *p* < 0.01, η^2^_p_= .347 |  |
| *24 hours* |  | *F*(1,24) = 10.043, *p* < 0.01, η^2^_p_= .295 |  | *F*(1,24) = 13.001, *p* = 0.001, η^2^_p_= .351 |  |
| *1 week* |  | *F*(1,24) = 30.718, *p* < 0.001, η^2^_p_= .561 |  | *F*(1,24) = 27.220, *p* < 0.001, η^2^_p_= .531 |  |
| *1 week*  *recognition test (d’)* |  | *F*(1,24) = 6.011, *p* < 0.05, η^2^_p_= .200 |  |  |  |
| ***Word retention (overall analysis)***  A mixed factors ANCOVA with within-subjects factor ‘delay’ (30min, 3h, 8h, and 24h), between-subjects factor ‘group’ (TEA patients versus Controls) and covariate “NART-predicted verbal IQ” revealed:  *Uncorrected data*: no significant effect of delay, *F*(3,72) = 0.895, *p* = 0.448, η^2^_p_= .036, a significant effect of group *F*(1,24) = 18.651, *p* < 0.001, η^2^_p_= .437, but no significant delay * group interaction: *F*(3,72) = 2.028, *p* = 0.118, η^2^_p_= .078.  *Guess-corrected data*: no significant effect of delay, *F*(3,72) = 1.285, *p* = 0.286, η^2^_p_= .051, a significant effect of group *F*(1,24) = 11.727, *p* < 0.01, η^2^_p_= .328, but no significant delay * group interaction: *F*(3,72) = 2.543, *p* = 0.063, η^2^_p_= .096, although the latter approached significance. | | | | | |
| ***Word retention (over which interval is ALF detectable)***  A mixed factors ANCOVA with within-subjects factor ‘delay’ (30min vs. 3h; 30min vs. 8h or 30min vs. 24h), between-subjects factor ‘group’ (TEA patients versus Controls) and covariate “NART-predicted verbal IQ” revealed the following interactions between delay and group: | | | | | |
|  |  | **Uncorrected data** |  | **Guess corrected data** |  |
|  |  | *TEA patients vs. controls* |  | *TEA patients vs. controls* |  |
| *30min – 3h interval* |  | *F*(1,24) = 0.802, *p* = 0.379, η^2^_p_= .032 |  | *F*(1,24) = 0.517, *p* = 0.479, η^2^_p_= .021 |  |
| *30min – 8h interval* |  | *F*(1,24) = 4.293, *p* < 0.05, η^2^_p_= .152 |  | *F*(1,24) = 4.583, *p* < 0.05, η^2^_p_= .160 |  |
| *30min – 24h interval* |  | *F*(1,24) = 4.389, *p* < 0.05, η^2^_p_= .155 |  | *F*(1,24) = 6.420, *p* < 0.05, η^2^_p_= .211 |  |
|  | | | | | |
